# Peristaltic transport characteristics of a second-grade dusty fluid flown with heat transfer through a tube revisited

**DOI:** 10.1038/s41598-022-22740-w

**Published:** 2022-12-14

**Authors:** N. M. Hafez, Reima D. Alsemiry, Sana A. Alharbi, A. M. Abd-Alla

**Affiliations:** 1 Department of Mathematics, Faculty of Education, Ain Shams University, Heliopolis (Roxy), Cairo, 11757 Egypt; 2 Department of Mathematics and Statistics, Faculty of Science, Taibah University, P.O. Box 89, Yanbu, 41911 Saudi Arabia; 3 Department of Mathematics, Faculty of Science, Sohag University, Sohag, Egypt

**Keywords:** Molecular medicine, Mathematics and computing

## Abstract

This paper provides a rudimentary insight into the influence of heat transfer on the transport characteristics of a second-grade dusty fluid flown in a flexible tube with walls subjected to the peristaltic motion. Both dust particles and fluid movements were modeled using the coupled differential equations. The effects of different types of parameters such as Reynolds number, Prandtl number, Grashof number, wave number, wave amplitude ratio, second grade parameter as well as nature of the heat source and sink are studies on the dust particles velocity, fluid velocity, temperature, pressure profiles of the fluid and streamline patterns of the fluid. The derived equations were solved analytically via the standard perturbation method to determine the fluid temperature, streamline pattern and velocity of the dust particles as well as fluid. The values in the increase of pressure and frictional forces were calculated numerically using DSolve of the Mathematica 11 software (https://www.wolfram.com/mathematica/new-in-11/). In addition, the trapping mechanisms were ascertained by computing the streamlines and various physical parameters. The obtained results were validated with the state-of-the-art literature reports. It was claimed that our systematic approach may constitute a basis for accurately examining the impact of heat transfer on the peristaltic transport of a complex fluid through narrow tubes, useful for diverse medical applications such as the gastric fluid flow through the small intestine during endoscopy. Numerical results are computed and discussed numerically and presented through graphs. The impacts of pertinent parameters on the aforementioned quantities are examined by plotting graphs on the basis of computational results. The results indicate that the effect of parameters is very pronounced. A suitable comparison has been made with the prior results in the literature as a limiting case of the considered problem.

## Introduction

Understanding the effects of heat transfer on the transport behavior of a second-grade dusty fluid streaming through a flexible tube with walls subjected to the peristaltic motion remains challenging^1^ . Generally, the perturbation approach is used to find the solution of such complex transport in various powers of the amplitude ratios^2^. It was discerned that for two-dimensional (2-D) peristaltic pumping with extremely small Reynolds numbers, the fluid motion can be assumed to be inertia-free and long wavelength in nature^3^. In addition, the peristaltic transport of different complex fluids in an axial-symmetric tube for certain values of the Reynolds number and wavelengths was studied wherein the asymptotic solutions were obtained concerning the small amplitudes to the mean diameter’s ratios^4^. This work was carried out following the earlier study on the fluid motion through 2-D channels^5^. Meanwhile, the long wavelength approximation for 2-D peristaltic pumping enabled in relaxing the assumption regarding the small amplitudes^6^. An all-inclusive overview on the peristaltic pumping was made by^7^.

The emergence of Navier Stokes relations for the rheological properties of fluids provided a further impetus to the advancement of the non-Newtonian fluid dynamics research^8^. The absence of unique constitutive model to describe the overall characteristics of the non-Newtonian fluids enforced the researchers to find out alternative models. To surmount this limitation, numerous classical models were introduced to obtain the nonlinear relationships among shear stresses and strain rates^9,10^. Generally, majority of the fluids including bio-fluids, synthetic lubricants, paints, oils, petroleum, honey, and so forth were shown to reveal the non-Newtonian traits^11^. Also, the mechanics of peristaltic pumping of a non-Newtonian fluid for a second-order fluid through an axisymmetric conduit was performed^12^. Over the years, several studies involving the non-Newtonian fluids have been conducted to determine the feasibility of practical applications in the field of physiology, engineering and industries^13–19^ .

Despite many dedicated efforts the behavior of pure fluids dynamics is far from being understood. Yet again, the natural fluids often contain various impurities and contaminants such as dirt and dust particles together with unknown components. The fluids are said to be dusty when they contain various dispersed solid particles in colloidal suspension, making the dynamics of such fluids very complex and yet worthy to investigate^20^. Examples of these dusty fluids include human urine with stones or glucose particles suspension, unrefined petroleum, crude oils and diverse foods containing pulpy granules. In the early period^21^, considered human blood as a binary system and analyzed its transport properties. Later^22^, examined the peristaltic transport behaviors of various dusty fluids under the approximation of long wavelength and small Reynolds numbers. Meanwhile^23^, investigated the transport properties of a solid fluid mix streaming through an axially-symmetric channel. Furthermore, the sinusoidal wave motion-induced 2-D flow of a dusty fluid moving through a tube with infinite curvy wall was examined for Reynolds numbers above one^24^. The peristaltic transport of a dusty fluid was demonstrated when streamed through a porous medium^25^. Recently^26^, evaluated the effects of the wall topologies of the channels on the transport attributes of tiny particles suspended in a dusty fluid. The impact of the wall topologies on the transport features of a Walters B fluid containing fine particulates flowing through a uniform channel was inspected^27^. The peristaltic transport characteristics of a dusty fluid streaming through a tube was demonstrated by^28^.

It is well established that the heat transfer is significant for sundry applications especially in the field of geophysical sciences and engineering like underground energy transport in the geothermal reservoirs, thermal insulation, porous solids’ drying, improvement in the recovery of fossil fuels and oils, catalytic reactors using packed-bed, and nuclear reactor’s cooling^29^. The influence of heat transfer on the peristaltic motion of a dusty fluid streaming through a channel was assessed^30^. In addition^31^, examined the effects of velocity slip on the magnetohydrodynamic (MHD) peristaltic transport of fluid through a porous media in the presence of both heat and mass transfer. Meanwhile^32^, determined the influence of rotating medium with compliant walls on the MHD fluid’s peristaltic transport. The dependence of temperatures on the peristaltic transport characteristics of a MHD fluid’s flowing through an asymmetric channel was analyzed^33^. The impact of compliant walls in the presence slip at the boundaries on the peristaltic transport behaviors of a MHD fluid was analytically formulated^34^. The influence of velocity slip on the MHD peristaltic transport of a Casson fluid and transfer of heat through an asymmetric channel cantaining a porous media was assessed^35^. Over a horizontal, flat plate with a constant heat flux, natural convection of power-law fluids was investigated^36^. Many authors have recently investigated the MHD convection for diffrent fluids under effect of heat transfer in the articles^37–39^ and^40,41^. The peristaltic transport features of a Johnson Segalman fluid flowing through an asymmetric curved channel accompanied by mass and heat transfer were analyzed^42^. A simulating sloshing and evaporation in a cryogenics fuel tank was studied using a new computational fluid dynamics algorithm^43^. A multinode-CFD simulation of a cylindrical pressurized cryogenic storage tank was examined by^44^. The study of cooling high temperature traveling wave tube collectors under steady state conditions with TWT collectors thermal stress and deformation was presented by^45^. Heat sink optimization and thermal performance enhancement in three geometry categories on energy storage system was discussed^46^.

Considering the immense fundamental and applied significance involving the impact of heat transfer on the transport behaviors of various dusty fluids streaming through a flexible tube with varying wall’s topologies subjected to the peristaltic motion, this paper considers a second-grade dusty fluid flowing in a flexible tube whose walls are induced by the peristaltic movement to understand the transport process in the presence of heat transfer. The influence of heat transfer on the peristaltic transport properties of such fluid was determined. The conventional perturbation theory was used to derive the analytical solution of the modeled coupled differential equations. The obtained results are validated with the solutions obtained by DSolve a built â€“ in function in Commerical software Mathematica 11 (https://www.wolfram.com/mathematica/new-in-11/). Therefore, the analytical solution has been obtained. The peristaltic transport behavior of the studied dusty fluid was evaluated in terms of the velocity of the dust particles and fluid, fluid temperature, friction force, pressure rise and streamline pattern. Furthermore, to ascertain the trapping process, different physical parameters and streamlines were computed and discussed by the graphical results. The obtained results were analyzed, discussed, and validated by comparing the outcomes of literature review^12^ and^20^. The peristaltic transport mechanism of the proposed dusty fluid was understood.

## Analytical model

**Figure 1:**
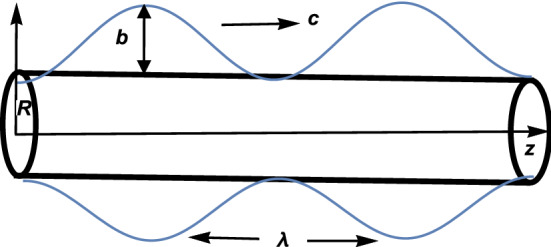
The tube geometry.

Consider an axial-symmetric 2-D streaming of a second-grade fluid through a tube enclosing tiny solid particles of even sizes with number density (*N*). The densities of the solid particles are constant. It is further assumed that long wavelength peristaltic waves can propagate along the tube walls. Let us assume the cylindrical coordinate system ${\overline{R}}({\overline{Z}}, {\overline{t}})$ with ${{\overline{R}}}$ and ${{\overline{Z}}}$ are along the radial and axial direction, respectively. Figure 1 shows a schematic diagram of the cylindrical wall described by:

1 $$\begin{aligned} {\overline{h}}({\overline{Z}},{\overline{t}})= a+b\;sin\frac{2 \pi }{\lambda }({{\overline{Z}}}-c{{\overline{t}}}) \end{aligned}$$

where *a*, *c*, ${\overline{t}}$, *b* and $\lambda$ are the radius of the tube, speed of propagating wave, time, wave amplitude and wavelength, respectively.

Following^47^, the stress tensor (constitutive relation) of the second-grade fluid can be written as:

2 $$\begin{aligned} \overline{{\varvec{S}}}&=-{{\overline{P}}}{\varvec{I}}+\varvec{\tau }, \end{aligned}$$

3 $$\begin{aligned} \overline{\varvec{\tau }}&=\mu \overline{\varvec{A_{1}}}+\alpha _{1} \overline{\varvec{A_{2}}}+\alpha _{2} (\bar{\varvec{A_{1}}})^{2}. \end{aligned}$$

where $\overline{\varvec{\tau }}$, $\mu$, $\alpha _{1}$ and $\alpha _{2}$; are the extra stress tensor, coefficient of viscosity and material constants. The kinematic tensors $\bar{\varvec{A_{1}}}$ and $\bar{\varvec{A_{2}} }$ can be written as^12^:

4 $$\begin{aligned} \bar{\varvec{A_{1}}}&= (\text {grad} {\bar{{\varvec{V}}}})+(\text {g}rad {\bar{{\varvec{V}}}})^{t}, \end{aligned}$$

5 $$\begin{aligned} \bar{\varvec{A_{2}}}&=\frac{d\bar{\varvec{A_{1}}}}{d\bar{t}} +\bar{\varvec{A_{1}}}(\text {grad} {\bar{{\varvec{V}}}})+(\text {grad} {\bar{{\varvec{V}}}})^{t} \bar{\varvec{A_{1}}} \end{aligned}$$

where grad and $\overline{{\varvec{V}}}=( {{\overline{U}}},0, \overline{W})$ are the gradient operator and velocity, respectively.

The equation of motion in the fixed frame $(\bar{R},\bar{Z})$ takes the form^28^:

6 $$\begin{aligned}&\rho [{\overline{U}} \frac{\partial {\overline{U}} }{\partial {\overline{R}}} + {\overline{W}} \frac{\partial {\overline{U}}}{\partial {\overline{Z}}}] \nonumber \\&\quad = -\frac{\partial {\overline{P}}}{\partial {\overline{R}}}+\frac{1}{{\overline{R}}} \frac{\partial }{\partial {\overline{Z}}} \left( {\overline{R}} \; {\overline{\tau }}_{rr}\right) + \frac{\partial }{\partial {\overline{Z}}}{\overline{\tau }} _{rz}\nonumber \\&\qquad - \frac{{\overline{\tau }}_{\theta \theta }}{{\overline{R}}} +K N ({\overline{u}} _{s}-{\overline{u}}), \end{aligned}$$

7 $$\begin{aligned}&\rho [{\overline{U}} \frac{\partial {\overline{W}}}{\partial {\overline{R}}} + {\overline{W}} \frac{\partial {\overline{W}}}{\partial {\overline{Z}}}] \nonumber \\&\quad = -\frac{\partial {\overline{P}}}{\partial {\overline{Z}}}+\frac{1}{{\overline{R}}} \frac{\partial }{\partial {\overline{R}}} \left( {\overline{R}}\;{\overline{\tau }} _{rz}\right) +\frac{\partial }{\partial {\overline{Z}}}{\overline{\tau }} _{zz}\nonumber \\&\qquad + K N ({\overline{W}}_{s}- {\overline{W}})-\rho g \alpha ({\overline{T}} -{\overline{T}}_{0}). \end{aligned}$$

The equation of motion for the solid particles^28^ and heat transfer are given by^15^:

8 $$\begin{aligned}&{\overline{U}}_{s} +\frac{\partial {\overline{U}}_{s}}{\partial {\overline{R}}}+ {\overline{W}}_{s} \frac{\partial {\overline{U}}_{s}}{\partial {\overline{Z}}} =\frac{K}{m} ({\overline{U}}-{\overline{U}}_{s}) \end{aligned}$$

9 $$\begin{aligned}&{\overline{U}}_{s} +\frac{\partial {\overline{W}}_{s}}{\partial {\overline{R}}}+ {\overline{W}}_{s} \frac{\partial {\overline{W}}_{s}}{\partial {\overline{Z}}} =\frac{K}{m} ({\overline{W}}-{\overline{W}}_{s}) \end{aligned}$$

10 $$\begin{aligned}&\rho c_{p}[{\overline{U}} \frac{\partial {\overline{T}}}{\partial {\overline{R}}}+ {\overline{W}}\frac{\partial {\overline{T}}}{\partial {\overline{Z}}}]=\kappa [\frac{\partial ^{2} {\overline{T}}}{\partial {\overline{R}}^{2}}+\frac{1}{{\overline{R}}} \frac{\partial {\overline{T}}}{\partial {\overline{R}}}+\frac{\partial ^{2} {\overline{T}}}{\partial {\overline{Z}}^{2} }]+Q_{0} \end{aligned}$$

where $\rho$, ${\overline{U}}$ and ${\overline{W}}$; ${\overline{U}}_{s}$ and ${\overline{W}}_{s}$ are the density, velocity components along the corresponding radial and axial direction of the fluid as well as dust particles motion, respectively; ${\overline{P}}$, *K*, *m*, *N*, *g*, $\alpha$, *T*, $c_{p}$, and $\kappa$ are the pressure, coefficient of resistance, mass, and number density (constant) of the solid dust particles; gravitational acceleration due to gravity, thermal expansion coefficient, temperature, specific heat at constant pressure, and thermal conductivity, respectively. The transformation corresponding to the moving and fixed reference frames $({\overline{r}},{\overline{z}})$ and $({\overline{R}}, {\overline{Z}})$ can be written as:

11 $$\begin{aligned}&{\overline{z}}={\overline{Z}}-c{\overline{t}},\; \overline{r}={\overline{R}},\; {\overline{w}}={\overline{W}}-c,\; {\overline{u}}=\overline{U},\;\nonumber \\&{\overline{w}}_{s}={\overline{W}}_{s}-c, \; {\overline{u}}_{s}={\overline{U}}_{s},\; {\overline{p}}={\overline{P}},\; {T}={\overline{T}} \end{aligned}$$

The flow Eqs. (6)–(10) are given by:

12 $$\begin{aligned} {\overline{u}} \frac{\partial {\overline{u}}}{\partial {\overline{r}}} + ({\overline{w}} + c) \frac{\partial {\overline{u}}}{\partial {\overline{z}}}&= -\frac{\partial {\overline{p}}}{\partial {\overline{r}}}+ \frac{1}{{\overline{r}}} \frac{\partial }{\partial {\overline{r}} }({\overline{r}}\; {\overline{\tau }}_{rr})+ \frac{\partial }{\partial {\overline{z}}}({\overline{r}}\; {\overline{\tau }}_{rz})- \frac{{\overline{\tau }}_{\theta \theta }}{{\overline{r}}}\nonumber \\&+ K N ({\overline{u}}_{s}-{\overline{u}}) \end{aligned}$$

13 $$\begin{aligned} {\overline{u}} \frac{\partial {\overline{w}}}{\partial {\overline{r}}} + ({\overline{w}} + c) \frac{\partial {\overline{w}}}{\partial {\overline{z}}}&=-\frac{\partial {\overline{p}}}{\partial {\overline{z}}}+ \frac{1}{{\overline{r}}} \frac{\partial }{\partial {\overline{r}} }({\overline{r}}\; {\overline{\tau }} _{rz})+\frac{\partial }{\partial {\overline{z}}} {\overline{\tau }}_{zz}+ K N ({\overline{w}}_{s}-{\overline{w}})\nonumber \\&+ \rho g \alpha ({\overline{T}}-{\overline{T}}_{0})\end{aligned}$$

14 $$\begin{aligned} {\bar{u}}_{s}\frac{\partial {\bar{u}}_{s}}{\partial {\bar{r}}}+ ( {\bar{w}}_{s}+c) \frac{\partial {\bar{u}}_{s}}{\partial {\bar{z}}}&= \frac{K}{m}(\bar{u}-\bar{u}_{s})\end{aligned}$$

15 $$\begin{aligned} {\bar{u}}_{s}\frac{\partial {\bar{w}}_{s}}{\partial {\bar{r}}}+ ({\bar{w}}_{s}+c) \frac{\partial {\bar{w}}_{s}}{\partial {\bar{z}}}&= \frac{K}{m}(\bar{w}-\bar{w}_{s})\end{aligned}$$

16 $$\begin{aligned} \rho C_{p}[{\overline{u}}\frac{\partial \overline{T}}{\partial {\overline{r}}}+ ({\overline{w}}+c)\frac{\partial {\overline{T}}}{\partial {\overline{z}}}]&=\kappa [\frac{\partial ^{2} {\overline{T}}}{\partial r^{2}}+\frac{1}{{\overline{r}}} \frac{\partial {\overline{T}}}{\partial {\overline{r}}}+\frac{\partial ^{2} {\overline{T}}}{\partial {\overline{z}}^{2}}]+Q_{0}. \end{aligned}$$

Let us introduce the following dimensionless variables and parameters:

17 $$\begin{aligned}&w=\frac{{{\overline{w}}}}{c},u=\frac{{{\overline{u}}}}{\delta c}, r=\frac{ {{\overline{r}}}}{a}, z=\frac{\overline{z}}{\lambda },{\overline{\tau }}=\frac{{\overline{\tau }}_{ij} a}{\mu c}, u_{s}=\frac{{{\overline{u}}}_{s}}{\delta c},\nonumber \\&w_{s}=\frac{{{\overline{w}}}_{s}}{c}, t=\frac{c {{\overline{t}}}}{\lambda } ,p=\frac{a^{2} {{\overline{p}}}}{\lambda \mu c},\theta = \frac{\overline{T}-{{\overline{T}}}_{0}}{\Delta \overline{T}},\delta =\frac{a}{\lambda },h=\frac{{{\overline{h}}}}{a}\nonumber \\&\text {Re}=\frac{\rho C a}{\mu }, \text {Pr} = \frac{\mu C_{p}}{k}, \text {Gr}=\frac{\rho g \alpha \Delta T a^{2}}{\mu c},\beta = \frac{Q_{0} a ^{2} }{k \Delta T},\nonumber \\&\alpha _{1}=\frac{\alpha _{1} c}{\mu a}, A= \frac{KNa^{2}}{\mu },B=\frac{K a}{m c}, \phi =\frac{b}{a} . \end{aligned}$$

where $\Delta {{\overline{T}}}={{\overline{T}}}_{1}-{{\overline{T}}}_{0}$ and ${{\overline{T}}}_{1}$, and ${{\overline{T}}}_{0}$ correspond to the temperature differences, upper and lower wall temperature. In addition, Re, Pr, Gr, and $\beta$ correspond to the Reynolds, Prandtl, Grashof number and parameter of heat source/sink.

Using Eq. (17), one obtains the following dimensionless form of Eqs. (12)–(16):

18 $$\begin{aligned} \delta ^{3} Re [ u \frac{\partial u}{\partial r}+ ( w+1) \frac{\partial u }{\partial z }]&= - \frac{\partial {\ {p}} }{\partial r}+ \frac{\delta }{ r}\frac{\partial }{\partial r}( r{\tau }_{rr})+\delta ^{2}\frac{\partial }{\partial {z}}{\tau _{rz}}\nonumber \\&\quad - \delta \frac{\tau _{\theta \theta }}{{r}} +\delta ^{2} A( u_{s} - u) \end{aligned}$$

19 $$\begin{aligned} \delta Re [{u} \frac{\partial w}{\partial r}+ ( w+1) \frac{\partial w }{\partial z }]&= - \frac{\partial {\ {p}} }{\partial z}- \frac{1}{ r} \frac{\partial }{\partial r}( r{\tau _{rz}})+\delta \frac{\partial }{\partial {z} }{\tau }_{zz}\nonumber \\&+A({w_{s}}-{w})- G r \theta \end{aligned}$$

20 $$\begin{aligned} \delta \left[ u_{s}\frac{\partial u_{s}}{\partial r}+ ( w_{s}+1) \frac{\partial u_{s}}{\partial z}\right]&= B( u- u_{s}) \end{aligned}$$

21 $$\begin{aligned} \delta \left[ u_{s}\frac{\partial w_{s}}{\partial r}+ (w_{s}+1) \frac{\partial w_{s}}{\partial z}\right]&= B( w- w_{s}) \end{aligned}$$

22 $$\begin{aligned} \delta \; \text {Re} \;\text {Pr} \big [u \frac{\partial \theta }{\partial r }+ (w + 1 )\frac{\partial \theta }{\partial z } \big ]&= \nabla ^{2} \theta + \beta \end{aligned}$$

Then, the dimensionless form of the stress tensor components are given by:

23 $$\begin{aligned} \tau _{rr}&= 2 \delta \frac{\partial u}{\partial r} + \alpha _{1} \big [(\frac{\partial w}{\partial r})^{2} - \delta ^{4} (\frac{\partial u}{\partial z})^{2} + 2 \delta ^{2} ( u \frac{\partial ^{2} u}{\partial r^{2}}+ w\frac{\partial ^{2} u}{\partial r \partial z})\big ] \end{aligned}$$

24 $$\begin{aligned} \tau _{rz}&=\delta ^{2}\frac{\partial u}{\partial z}+\frac{\partial w}{\partial r} + \alpha _{1} \big [\delta ^{3} \frac{\partial u}{\partial r} \frac{\partial u}{\partial z}+ \delta \frac{\partial w}{\partial r} \frac{\partial w}{\partial z} -\delta \frac{\partial u}{\partial r} \frac{\partial w}{\partial r} -\delta ^{3}\frac{\partial u}{\partial z} \frac{\partial w}{\partial z}\nonumber \\&\quad +\delta ^{3} u \frac{\partial ^{2}u}{\partial r \partial z}+ \delta u \frac{\partial ^{2} w}{\partial r^{2}}+\delta ^{3} w\frac{\partial ^{2} u}{\partial z^{2}}+\delta w \frac{\partial ^{2} w}{\partial r\partial z}\big ] \end{aligned}$$

25 $$\begin{aligned} \tau _{zz}&= 2 \delta \frac{\partial w}{\partial z} +\alpha _{1} \big [\delta ^{4}(\frac{\partial u}{\partial z})^{2}- (\frac{\partial w}{\partial r} )^{2}+2\delta \big [ u \frac{\partial ^{2}w}{\partial r \partial z}+ w\frac{\partial ^{2} w}{\partial z^{2}}\big ]\end{aligned}$$

26 $$\begin{aligned} \tau _{\theta \theta }&=2 \delta \frac{u}{r}+2\alpha _{1} \delta ^{2}\big [\frac{u}{r}\frac{\partial u}{\partial r}-\frac{u^{2}}{r^{2}}+\frac{w}{r} \frac{\partial u}{\partial z}\big ] \end{aligned}$$

with dimensionless $\nabla ^{2}$ of the form:

27 $$\begin{aligned} \nabla ^{2}= \frac{\partial ^{2}}{\partial r^{2}}+\frac{1}{r}\frac{\partial }{\partial r}+\delta ^{2} \frac{\partial ^{2}}{\partial z^{2}} \end{aligned}$$

The flow equations can be expressed as:

28 $$\begin{aligned}&u=-\frac{1}{r}\frac{\partial \psi }{\partial z},w=\frac{1}{r}\frac{\partial \psi }{\partial r}, \end{aligned}$$

29 $$\begin{aligned}&u_{s}=-\frac{1}{r}\frac{\partial \varphi }{\partial z},w_{s}=\frac{1}{r} \frac{\partial \varphi }{\partial r}. \end{aligned}$$

By combining Eqs. (28) and (29) with Eqs. (18) and (26) one achieves:

30 $$\begin{aligned}&\text {Re}\;\delta \bigg \{\delta ^{2} \big [\frac{-2}{r^{2}}\frac{\partial \psi }{\partial z}\frac{\partial ^{2} \psi }{\partial z^{2}} +\frac{1}{r} \frac{\partial ^{3} \psi }{\partial z^{3}}\frac{\partial \psi }{\partial r} -\frac{1}{r}\frac{\partial ^{3}}{\partial ^{3}}\frac{\partial \psi }{\partial r}-\frac{\partial ^{3}\psi }{\partial z^{3}} \big ]\nonumber \\&\qquad -\big [-\frac{3}{r^{3}}\frac{\partial \psi }{\partial r}\frac{\partial \psi }{\partial z}+\frac{3}{r^{2}}\frac{\partial \psi }{\partial z}\frac{\partial ^{2}\psi }{\partial r^{2}}-\frac{1}{r}\frac{\partial }{\partial \psi } \frac{\partial ^{3}\psi }{\partial r^{3}}-\frac{1}{r^{2}}\frac{\partial \psi }{\partial r}\frac{\partial ^{2}\psi }{\partial r \partial z}\nonumber \\&\qquad +\frac{1}{r}\frac{\partial \psi }{\partial r}\frac{\partial ^{3}\psi }{\partial r^{2} \partial z}+\frac{1}{r}\frac{\partial ^{2}\psi }{\partial r\partial z}+\frac{\partial ^{3}\psi }{\partial r^{2}\partial z}\big ]\bigg \}\nonumber \\&\quad =\delta \frac{\partial ^{2}}{\partial r\partial z}(r \tau _{rr})+\delta ^{2} r\frac{\partial ^{2} \tau _{rz}}{\partial z^{2}}-\delta \frac{\partial ^{2} \tau _{\theta \theta }}{\partial z}\nonumber \\&\qquad -r\frac{\partial }{\partial r}(\frac{1}{r} \frac{\partial }{\partial r} \tau _{rz})-\delta r\frac{\partial ^{2}\tau _{zz}}{\partial r\partial z} -A(\nabla _{1}^{2} \varphi -\nabla _{1}^{2}\psi )\nonumber \\&\qquad - \text {Gr} r \frac{\partial \theta }{\partial r} \end{aligned}$$

31 $$\begin{aligned}&\delta \bigg \{ \delta ^{2} \big [-\frac{2}{r^{2}}\frac{\partial \varphi }{\partial z}\frac{\partial ^{2} \varphi }{\partial z^{2}}+ \frac{1}{r} \frac{\partial \varphi }{\partial z}\frac{\partial ^{3} \varphi }{\partial r \partial z^{2}}-\frac{1}{r}\frac{\partial ^{3} \varphi }{\partial z^{3}} \frac{\partial \varphi }{\partial r} - \frac{\partial ^{3} \varphi }{\partial z^{3}}\big ]\nonumber \\&\qquad - \big [-\frac{3}{r^{3}}\frac{\partial \varphi }{\partial r}\frac{\partial \varphi }{\partial z}+ \frac{3}{r^{2}}\frac{\partial ^{2} \varphi }{\partial r^{2}}\frac{\partial \varphi }{\partial z} -\frac{1}{r^{2}} \frac{\partial ^{2} \varphi }{\partial r\partial z }\frac{\partial \varphi }{\partial r}- \frac{1}{r}\frac{\partial ^{3} \varphi }{\partial r^{3}} \frac{\partial \varphi }{\partial z}\nonumber \\&\qquad + \frac{1}{r}\frac{\partial ^{3} \varphi }{\partial r^{2}\partial z} \frac{\partial \varphi }{\partial z}- \frac{1}{r}\frac{\partial ^{2} \varphi }{\partial r\partial z}+\frac{\partial ^{3} \varphi }{\partial r^{2}\partial z}\big ]\bigg \}= B(\nabla ^{2}_{1} \varphi -\nabla _{1}^{2} \psi ) \end{aligned}$$

The dimensionless time flow relations for the fluid and the solid particles take the form:

$$\begin{aligned} Q&= F + \frac{1}{2}( 1+ \phi \sin z)^{2}\\ Q_{s}&= F_{s} + \frac{1}{2}( 1+ \phi \sin z)^{2} \end{aligned}$$

where

32 $$\begin{aligned} F&= \int _{0}^{h}\frac{\partial \psi }{\partial r} dr= \psi (h)-\psi (0) \end{aligned}$$

33 $$\begin{aligned} F_{s}&= \int _{0}^{h}\frac{\partial \varphi }{\partial r} dr= \varphi (h)-\varphi (0) \end{aligned}$$

The expression for the pressure rise $\Delta {p}$ and the friction force $F_{\lambda }$, respectively can be written as:

$$\begin{aligned} \Delta {p}=\int _{0}^{2\pi }\frac{d p}{d z} dz \end{aligned}$$

and

$$\begin{aligned} F_{\lambda }=\int _{0}^{2\pi }h^{2}(-\frac{d p}{d z}) dz \end{aligned}$$

where

$$\begin{aligned} h=1+\phi \sin z \end{aligned}$$

From Eq. (19) one gets $\frac{d p}{d z}$ in the form of:

34 $$\begin{aligned} \frac{d p}{d z}&=\frac{1}{r}\frac{\partial }{\partial r}(r \tau _{r}z)+\delta \frac{\partial }{\partial z}(tau_{z}z)+\frac{A}{r} (\frac{\partial \varphi }{\partial r}-\frac{\partial \psi }{\partial r})\nonumber \\&\quad +\text {Gr}\theta -\delta \text {Re}\bigg [-\frac{1}{r}\frac{\partial \psi }{\partial z}\frac{\partial }{\partial r}(\frac{1}{r}\frac{\partial \psi }{\partial r})\nonumber \\&\quad +(\frac{1}{r}\frac{\partial \psi }{\partial r}+1)\frac{\partial }{\partial z}(\frac{1}{r}\frac{\partial \psi }{\partial r}) \bigg ] \end{aligned}$$

In the dimensionless wave frame, the boundary conditions can be casted as:

35 $$\begin{aligned}&\psi =0,\; \varphi =0,\; \frac{\partial }{\partial r}(\frac{1}{r}\frac{\partial \psi }{\partial r})=0 \text{ at } r=0 \end{aligned}$$

36 $$\begin{aligned}&\psi =F,\; \varphi =F_{s},\; \frac{1}{r}\frac{\partial \psi }{\partial r}=-1 \text{ at } r=h \end{aligned}$$

37 $$\begin{aligned}&\frac{\partial \theta }{\partial r}=0 \text{ at } r=0 \end{aligned}$$

38 $$\begin{aligned}&\theta =0 \text{ at } r=h \end{aligned}$$

## Analytical method

Following the perturbation procedure to introduce the physical properties $\psi ,\phi ,\theta ,F,F_{s}$ and *p* as a mathematically power series with small perturbation parameter $\delta$ we then substitute into the governing Eqs. (30–34) to obtain three sets of equations and boundary conditions (35–38) resulting from collecting the similar power terms $\delta$ and $\delta ^{2}$ which are sufficient for computing the solution. The Eqs. (30–34) subjected to the boundary conditions (35–38) have been solved analytically by the perturbation procedure, formally known described in^25^.

## Analytical solutions

As aforementioned, the perturbation technique was used to get the solutions of a set of nonlinear equations of the fluid and dust particles derived from the analytical model presented in the last section. Following the method referred in^25^, in terms of a small perturbation parameter $\delta$ one obtains various perturbation series as:

39 $$\begin{aligned} \psi&=\psi _{0} + \delta \psi _{1}+ \delta ^{2} \psi _{2}+ O(\delta ^{3}); \end{aligned}$$

40 $$\begin{aligned} \varphi&=\varphi _{0} + \delta \varphi _{1}+ \delta ^{2}\varphi _{2}+ O(\delta ^{3});\end{aligned}$$

41 $$\begin{aligned} \theta&=\theta _{0} + \delta \theta _{1}+ \delta ^{2}\theta _{2}+ O(\delta ^{3});\end{aligned}$$

42 $$\begin{aligned} F&=F_{0} + \delta F_{1}+ \delta ^{2} F_{2}+ O(\delta ^{3});\end{aligned}$$

43 $$\begin{aligned} F_{s}&=F_{s_{0}} + \delta F_{s_{1}}+ \delta ^{2}F_{s_{2}}+ O(\delta ^{3});\end{aligned}$$

44 $$\begin{aligned} p&=p_{0} + \delta p_{1}+ \delta ^{2}p_{2}+ O(\delta ^{3}) . \end{aligned}$$

By combining Eqs. (39)–(44), (30)–(34) and (35)–(38) one gets the underlying zeroth-order, first-order and second-order equations together with the corresponding boundary conditions given by:

### zero-Order Solution of O($\delta$)

45 $$\begin{aligned}&-r \frac{\partial }{\partial r}\big [\frac{1}{r}\frac{\partial }{\partial r}( r \tau _{0rz})\big ] - A\big [\frac{\partial ^{2}}{\partial r^{2}}-\frac{1}{r} \frac{\partial }{\partial r}\big ](\varphi _{0}-\psi _{0}) - \text {Gr} r \frac{\partial \theta _{0}}{\partial r} =0 \end{aligned}$$

46 $$\begin{aligned}&B\big [\frac{\partial ^{2}}{\partial r^{2}}- \frac{1}{r}\frac{\partial }{\partial r}\big ](\varphi _{0}-\psi _{0})=0 \end{aligned}$$

47 $$\begin{aligned}&\big [\frac{\partial ^{2}}{\partial r^{2}}- \frac{1}{r}\frac{\partial }{\partial r}\big ]\theta _{0}+\beta =0 \end{aligned}$$

48 $$\begin{aligned}&\frac{d {\ {p_{0}}} }{d z} = \frac{1}{ r}\frac{\partial }{\partial r}( r{\tau _{0rz}})+\frac{A}{r}(\frac{\partial \varphi _{0}}{\partial r} -\frac{\partial \psi _{0}}{\partial r})+\text {Gr}\theta _{0} \end{aligned}$$

where

$$\begin{aligned} \tau _{0rz}=\frac{\partial w_{0}}{\partial r}=\frac{\partial }{\partial r} (\frac{1}{r}\frac{\partial \psi _{0}}{\partial r}) \end{aligned}$$

with the boundary conditions

49 $$\begin{aligned}&\psi _{0}=0, \;\;\frac{\partial }{\partial r}(\frac{1}{r}\frac{\partial \psi _{0}}{\partial r})=0,\;\;\frac{\partial \theta _{0}}{\partial r} =0,\;\;\varphi _{0}=0\;\; \text {on}\; r=0 \end{aligned}$$

50 $$\begin{aligned}&\psi _{0}=F_{0},\;\; \frac{1}{r}\frac{\partial \psi _{0}}{\partial r}=0,\;\; \theta _{0}=0,\;\;\varphi _{0}= F_{s_{0}}\;\;\text {on}\; r=h \end{aligned}$$

Substituting Eqs. (49)–(50) into the Eqs. (45)–(48) one obtains:

51 $$\begin{aligned} \psi _{0}(r,z)&=\frac{1}{384} r^{2} \left( 96 \left( r^{2} C_{3}(z)+2 C_{1}(z)\right) +\beta \text {Gr} r^{4}\right) \end{aligned}$$

52 $$\begin{aligned} \varphi _{0}(r,z)&=\frac{1}{4} r^{4} C_{3}(z)+\frac{1}{2} r^{2} C_{4}(z)+\frac{1}{384} \beta \text {Gr} r^{6} \end{aligned}$$

53 $$\begin{aligned} \theta _{0}(r, z)&= -(r^{2} \beta )/4) + A_{1} \end{aligned}$$

Where the constants $A_{1}, C_{1},C_{3}, C_{4}$ are expressed as:

54 $$\begin{aligned} A_{1}&=\frac{h^{2} \beta }{4}; \end{aligned}$$

55 $$\begin{aligned} C_{1}&=\frac{4 F_{0}}{h^{2}}+\frac{1}{192} \beta \text {Gr} h^{4}+1;\end{aligned}$$

56 $$\begin{aligned} C_{3}&=-\frac{2}{h^{2}}-\frac{1}{48} \text {Gr} h^{2} \beta - \frac{4 F_{0} }{h^{4}};\end{aligned}$$

57 $$\begin{aligned} C_{4}&= 1 + \frac{1}{192} \text {Gr}; h^{4} \beta + \frac{2 (F_{0}+F_{s0} )}{h^{2}}. \end{aligned}$$

### first-Order Solution of O($\delta$)

The first-order approximation of O($\delta$)attains the following equations:

58 $$\begin{aligned}&\text {Re} \Bigg [ \frac{3}{r^{3}}\frac{\partial \psi _{0}}{\partial r} \frac{\partial \psi _{0}}{\partial z} +\frac{3}{r^{2}}\frac{\partial \psi _{0} }{\partial z}\frac{\partial ^{2}\psi _{0}}{\partial r^{2}}+\frac{1}{r} \frac{\partial \psi _{0}}{\partial z}\frac{\partial ^{3}\psi _{0}}{\partial r^{3} }\nonumber \\&\qquad +\frac{1}{r^{2}}\frac{\partial \psi _{0}}{\partial r}\frac{\partial ^{2} \psi _{0}}{\partial r\partial z}-\frac{1}{r}\frac{\partial \psi _{0}}{\partial z}\frac{\partial ^{3}\psi _{0}}{\partial r^{2}\partial z}+\frac{1}{r} \frac{\partial ^{2} \psi _{0}}{\partial r\partial z}- \frac{\partial ^{3}\psi _{0}}{\partial r^{2}\partial z}\Bigg ]\nonumber \\&\quad =\frac{\partial ^{2}}{\partial r \partial z}(r \tau _{0rr})- r \frac{\partial }{\partial r}\bigg [\frac{1}{r}\frac{\partial }{\partial r}(r \tau _{1rz})\bigg ]-r \frac{\partial ^{2}\tau _{0zz}}{\partial r \partial z}\nonumber \\&\qquad -A(\nabla ^{2} \varphi _{1} - \nabla ^{2}_{1}\psi _{1})- \text {Gr} r\frac{\partial \theta _{1}}{\partial r} \end{aligned}$$

59 $$\begin{aligned}&\frac{3}{r^{3}}\frac{\partial \varphi _{0}}{\partial r}\frac{\partial \varphi _{0}}{\partial z}-\frac{3}{r^{2}}\frac{\partial ^{2}\varphi _{0} }{\partial r^{2}}\frac{\partial \varphi _{0}}{\partial z}+\frac{1}{r^{2}} \frac{\partial \varphi _{0}}{\partial r}\frac{\partial \varphi _{0}}{\partial r}\frac{\partial ^{2} \varphi _{0}}{\partial r\partial z}\nonumber \\&\qquad + \frac{1}{r}\frac{\partial ^{3}\varphi _{1}}{\partial r^{3}}\frac{\partial \varphi _{0}}{\partial z}-\frac{1}{r}\frac{\partial ^{2}\varphi _{0} }{\partial r\partial z}\frac{\partial \varphi _{0}}{\partial r}+\frac{1}{r} \frac{\partial ^{2}\varphi _{0}}{\partial r\partial z}-\frac{\partial ^{3} \varphi _{0}}{\partial r^{2}\partial z}\nonumber \\&\quad =B(\nabla ^{2}_{1} \varphi _{1} - \nabla ^{2}_{1}\psi _{1}) \end{aligned}$$

60 $$\begin{aligned}&\big (\frac{\partial ^{2}}{\partial r^{2}}-\frac{1}{r}\frac{\partial }{\partial r}\big )\theta _{1}=\text {Re}\text {Pr}\bigg [-\frac{1}{r}\frac{\partial \psi _{0} }{\partial z}\frac{\partial \theta _{0}}{\partial r}+ ( \frac{1}{r} \frac{\partial \psi _{0}}{\partial r}+1)\frac{\partial \theta _{0}}{\partial z}\bigg ] \end{aligned}$$

61 $$\begin{aligned}&\frac{d {\ {p_{1}}} }{d z} = \frac{1}{ r}\frac{\partial }{\partial r}( r{\tau _{1rz}})+\frac{\partial }{\partial z}( {\tau _{0zz}})+ \frac{A}{r} (\frac{\partial \varphi _{1}}{\partial r}-\frac{\partial \psi _{1}}{\partial r})+\text {Gr}\theta _{1}\nonumber \\&\qquad -\text {Re} \bigg [-\frac{1}{ r}\frac{\partial \psi _{0}}{\partial r} \frac{\partial }{\partial r}(\frac{1}{ r}\frac{\partial \psi _{0}}{\partial r})+(\frac{1}{ r}\frac{\partial \psi _{0}}{\partial r}+1)\frac{\partial }{\partial z}(\frac{1}{ r}\frac{\partial \psi _{0}}{\partial r}) \bigg ] \end{aligned}$$

The boundary conditions are written as:

62 $$\begin{aligned}&\psi _{1}=0, \;\;\frac{\partial }{\partial r}(\frac{1}{r}\frac{\partial \psi _{1}}{\partial r})=0,\;\;\frac{\partial \theta _{1}}{\partial r} =0,\;\;\varphi _{0}=0\;\; \text {on}\; r=0 \end{aligned}$$

63 $$\begin{aligned}&\psi _{1}=F_{1},\;\; \frac{1}{r}\frac{\partial \psi _{1}}{\partial r}=0,\;\; \theta _{1}=0,\;\;\varphi _{1}= F_{s_{1}}\;\; \text {on}\; r=h \end{aligned}$$

where

64 $$\begin{aligned} \tau _{0rr}&=\alpha _{1} \left( \frac{\partial }{\partial r}\bigg ( \frac{1}{r}\frac{\partial \psi _{0}(r,z)}{\partial r}\bigg )\right) ^{2} \end{aligned}$$

65 $$\begin{aligned} \tau _{1rz}&=\frac{\partial }{\partial r}(\frac{1}{r} \frac{\partial \psi _{1} }{\partial r})-\frac{1}{r}\frac{\partial ^{2}\psi _{0}}{\partial z^{2}} +\alpha _{1}\Bigg [\frac{\partial }{\partial r}(\frac{1}{r}\frac{\partial \psi _{0}}{\partial r})\frac{1}{r}\frac{\partial ^{2} \psi _{0}}{\partial r\partial z}\nonumber \\&\quad +\frac{\partial }{\partial r}(\frac{1}{r}\frac{\partial \psi _{0}}{\partial r})\frac{1}{r}\frac{\partial ^{2} \psi _{0}}{\partial r\partial z} -\frac{1}{r}\frac{\partial \psi _{0}}{\partial z}\frac{\partial ^{2}}{\partial r^{2} }(\frac{1}{r}\frac{\partial \psi _{0}}{\partial r})\nonumber \\&\quad +\frac{1}{r}\frac{\partial \psi _{0}}{\partial r}\frac{\partial ^{2}}{\partial r\partial z}(\frac{1}{r}\frac{\partial \psi _{0}}{\partial r})\Bigg ] \end{aligned}$$

66 $$\begin{aligned} \tau _{0zz}&=-\alpha _{1}\Bigg (\frac{\partial }{\partial r}\big (\frac{1}{r} \frac{\partial \psi _{1}}{\partial r}\big ) \Bigg )^{2} \end{aligned}$$

Through the algebraic manipulations of the Eqs. (51)–(53), (58)–(61), and (62)–(63) one attains the solution of the First-Order equations of the the form:

67 $$\begin{aligned} \psi _{1}(r,z) =&-\frac{1}{361267200 B} \Bigg (245 B \text {Gr}\; \text {Pr} r^{6} R \bigg (3840 + \text {Gr} r^{4} \beta + 3840 C_{1}\nonumber \\&+320 r^{2} C_{3}\bigg )A_{1}^{^{\prime }} +8\bigg (2450 B Gr (1 + Pr) r^{8} R \beta C_{1}^{^{\prime }}+ r^{6} (-470400\nonumber \\&*(A + B R) + 245 B \text {Gr} \text {Pr} r^{4} R \beta -16 (B \alpha _{1} (25 \text {Gr} r^{2} (-343 + 64 r) \beta \nonumber \\&+ 5376 (-175 + 24 r) C_{3}) +4900 (6 B R C_{1} + r^{2} (A + B R) C_{3}\nonumber \\&+ 6 A C_{4}))) C_{3}^{^{\prime }} +2450(-4608 B r^{2} \left( r^{2} 2C_{3} ^{*}+2 C_{1}^{*}\right) +A Gr r^{8} \beta C_{4}^{^{\prime }} )\bigg )\Bigg ) \end{aligned}$$

68 $$\begin{aligned} \varphi _{1}(r,z) =&\Bigg ( 245 B \text {Gr} \text {Pr} r^{6} R (3840 + \text {Gr} r^{4} \beta + 3840 +C_{1} 320 r^{2} C_{3}) A_{1}^{^{\prime } }\nonumber \\&+8\bigg (2450 B \text {Gr} (1 + \text {Pr}) r^{8} R \beta C_{1}^{^{\prime }} +r^{4}(16 \alpha _{1} B r^{2} (25 \beta \text {Gr} (343-64 r) r^{2}\nonumber \\&-5376 (24 r-175) C_{3}) +245(-1920 (-24 + r^{2} (A + B R)) + B \text {Gr} \text {Pr} r^{6} R \beta \nonumber \\&+ 46080 C_{4}-320 r^{2} (C_{3} (r^{2} (A+B R)-48)+6 A C_{4}+6 B R C_{1})))C_{3}^{^{\prime }}\nonumber \\&+2450 (\beta \text {Gr} r^{6} (A r^{2}-48) C_{4}^{^{\prime }} -4608 B(r^{4} C_{3}^{*}+2 r^{2} C_{4}^{*}))\bigg )\Bigg ) \end{aligned}$$

69 $$\begin{aligned} \theta _{1}(r,z) =&\frac{\text {Pr} r^{2} R}{{2304}} \Bigg (A_{1}^{^{\prime }} \left( 144 r^{2} C_{3}+576 C_{1}+\beta \text {Gr} r^{4}+576\right) +4 \beta r^{2} (2 r^{2} C_{3}^{\prime }(z)\nonumber \\&+9 C_{1}{^{\prime }})\Bigg ) +A_{2} \end{aligned}$$

where the constants $C_{1}^{*},C_{3}^{*}, C_{4}^{*}, A_{2}^{^{\prime }}$ are defined as:

70 $$\begin{aligned} C_{1}^{*} =&\frac{1}{180633600 B h^{2}}\Bigg ( 722534400 B F_{1}-h^{6} (8 (-C_{3}^{\prime }(32 (4900\nonumber \\&\times (h^{2} C_{3} (A+B R)+3 A C_{4}(z)+3 B R C_{1}) +\alpha _{1} B (2688 (36 h\nonumber \\&-175) C_{3} +25 \beta \text {Gr} (80 h-343) h^{2}))+735 (640 (A+B R)\nonumber \\&-\beta B \text {Gr} h^{4} \Pr R)) +4900 A \beta \text {Gr} h^{2} C_{4}^{\prime }+4900 \beta B \text {Gr} h^{2}\nonumber \\&\times (\Pr +1) R C_{1}^{\prime }) +245 B \text {Gr} \Pr R A_{1}{^{\prime } }\nonumber \\&\times (640 h^{2} C_{3}+3840 C_{1}+3 \beta \text {Gr} h^{4}+3840))\Bigg ) \end{aligned}$$

71 $$\begin{aligned} C_{3}^{*} =&\frac{1}{645120 B h^{4}}\Bigg ( h^{6} (4 (-2 C_{3}^{\prime }(4 (420 (h^{2} C_{3} (A+B R)+4 A C_{4}+4 B R C_{1})\nonumber \\&+\alpha _{1} B (1536 (6 h-35) C_{3}+5 \beta \text {Gr} (32 h-147) h^{2}))\nonumber \\&+6720 (A+B R)-7 \beta B \text {Gr} h^{4} \text {Pr} R)+105 A \beta \text {Gr} h^{2} C_{4}^{\prime }\nonumber \\&+105 \beta B \text {Gr} h^{2} (\text {Pr} +1) R C_{1}^{\prime }(z))+7 B \text {Gr} \Pr R A_{1}^{\prime }(240 h^{2} C_{3}+1920 C_{1}\nonumber \\&+\beta \text {Gr} h^{4}+1920))-2580480 B F_{1}\Bigg ) \end{aligned}$$

72 $$\begin{aligned} C_{4}^{*}=&\frac{2}{h^{2}}\Bigg (F_{s1}+ \frac{1}{361267200 B}\bigg ( h^{4} (8 (2450 (\beta \text {Gr} h^{2} (A h^{2}-48) C_{4}^{\prime }\nonumber \\&-4608 B C_{3}{}^{*})-C_{3}^{\prime }(16 h^{2} (4900 (C_{3} (h^{2} (A+B R)-48)+6 A C_{4})+6 B R C_{1})\nonumber \\&+ \alpha _{1} B (5376 (24 h-175) C_{3}+25 \beta \text {Gr} (64 h-343) h^{2}))\nonumber \\&+245 (1920 (h^{2} (A+B R)-24)+\beta (-B) \text {Gr} h^{6} \text {Pr} R-46080 C_{4}))\nonumber \\&+2450 \beta B \text {Gr} h^{4} (\text {Pr} +1) R C_{1}^{\prime })+245 B \text {Gr} h^{2} \text {Pr} R A_{1}^{\prime }(320 h^{2} C_{3}+3840 C_{1} \nonumber \\&+\beta \text {Gr} h^{4}+3840)\bigg )\Bigg ) \end{aligned}$$

73 $$\begin{aligned} A_{2}^{^{\prime }}=&\frac{-h^{2} \text {Pr} R}{2304}\Bigg ( A_{1}^{\prime }(144 h^{2} C_{3}+576 C_{1}+\beta \text {Gr} h^{4}+576)+4 \beta h^{2} (2 h^{2} C_{3}^{\prime }+9 C_{1}^{\prime })\Bigg ) \end{aligned}$$

### second-Order Solution of O($\delta ^{2}$)

Write few lines prelude here to make this mathematical machinery meaningful and the purpose of these steps:

74 $$\begin{aligned}&\text { Re} \Bigg [\frac{3}{r^{3}}(\frac{\partial \psi _{0}}{\partial r} \frac{\partial \psi _{1}}{\partial z}+\frac{\partial \psi _{1}}{\partial r} \frac{\partial \psi _{0}}{\partial z}) -\frac{3}{r^{2}}(\frac{\partial \psi _{0} }{\partial z}\frac{\partial ^{2}\psi _{1}}{\partial r^{2}}+\frac{\partial \psi _{1}}{\partial z}\frac{\partial ^{2}\psi _{0}}{\partial r^{2}})\nonumber \\&\qquad +\frac{1}{r}(\frac{\partial \psi _{0}}{\partial z}\frac{\partial ^{3}\psi _{1} }{\partial r^{3}}+\frac{\partial \psi _{1}}{\partial z}\frac{\partial ^{3} \psi _{0}}{\partial r^{3}}) +\frac{1}{r^{2}}(\frac{\partial \psi _{0}}{\partial r}\frac{\partial ^{2}\psi _{1}}{\partial r\partial z}+\frac{\partial \psi _{1} }{\partial r}\frac{\partial ^{2}\psi _{0}}{\partial r\partial z})\nonumber \\&\qquad -\frac{1}{r}(\frac{\partial \psi _{0}}{\partial z}\frac{\partial ^{3}\psi _{1} }{\partial r^{2}\partial z}+\frac{\partial \psi _{1}}{\partial r}\frac{\partial ^{3}\psi _{0}}{\partial r^{2}\partial z}) +\frac{1}{r}(\frac{\partial ^{2} \psi _{1}}{\partial r\partial z})- \frac{\partial ^{3}\psi _{1} }{\partial r^{2}\partial z}\Bigg ]\nonumber \\&\quad =\frac{\partial ^{2}}{\partial r \partial z}(r T_{1rr})+r^{2}\frac{\partial ^{2}}{\partial z^{2}} (T_{0rz})-\frac{\partial T_{1\theta \theta } }{\partial z}-r\frac{\partial }{\partial r}[\frac{1}{r}\frac{\partial }{\partial r}(rT_{2rz})]\nonumber \\&\qquad -r\frac{\partial ^{2}T_{1zz}}{\partial r\partial z}-A(\frac{\partial ^{2} }{\partial r^{2}} -\frac{1}{r}\frac{\partial }{\partial r})(\varphi _{2} -\psi _{2})-A \frac{\partial ^{2}}{\partial z^{2}} (\varphi _{0}-\psi _{0})\nonumber \\&\qquad - G_{r}r\frac{\partial \theta _{2}}{\partial r} \end{aligned}$$

75 $$\begin{aligned}&\frac{3}{r^{3}}\Bigg [\frac{\partial \varphi _{0}}{\partial r}\frac{\partial \varphi _{1}}{\partial z}+\frac{\partial \varphi _{1}}{\partial r} \frac{\partial \varphi _{0}}{\partial z}\Bigg ]-\frac{3}{r^{2}}\Bigg [\frac{\partial \varphi _{1}}{\partial z}\frac{\partial ^{2}\varphi _{0}}{\partial r^{2}}+\frac{\partial \varphi _{0}}{\partial z}\frac{\partial ^{2}\varphi _{1} }{\partial r^{2}}\Bigg ]\nonumber \\&\qquad +\frac{1}{r^{2}}\Bigg [\frac{\partial \varphi _{1}}{\partial r}\frac{\partial ^{2}\varphi _{0}}{\partial r\partial z}+\frac{\partial \varphi _{0} }{\partial r}\frac{\partial ^{2}\varphi _{1}}{\partial r\partial z} \Bigg ]+\frac{1}{r}\Bigg [\frac{\partial \varphi _{1}}{\partial z}\frac{\partial ^{3}\varphi _{0}}{\partial r^{3}}+\frac{\partial \varphi _{0}}{\partial z}\frac{\partial ^{3}\varphi _{1}}{\partial r^{3}}\Bigg ]\nonumber \\&\qquad -\frac{1}{r}\Bigg [\frac{\partial \varphi _{1}}{\partial z}\frac{\partial ^{3}\varphi _{0}}{\partial r^{2}\partial z}+\frac{\partial \varphi _{0}}{\partial r}\frac{\partial ^{3}\varphi _{1}}{\partial r^{2}\partial z}\Bigg ]+\frac{1}{r}(\frac{\partial ^{2}\varphi _{1}}{\partial r\partial z})-\frac{\partial ^{3}\varphi _{1}}{\partial r^{2}\partial z}\nonumber \\&\quad =B(\frac{\partial ^{2}}{\partial r^{2}}-\frac{1}{r}\frac{\partial }{\partial r})(\varphi _{2}-\psi _{2})+B\frac{\partial ^{2}}{\partial z^{2}}(\varphi _{0}-\psi _{0}) \end{aligned}$$

76 $$\begin{aligned}&\frac{\partial ^{2}\theta _{2}}{\partial r^{2}}+\frac{1}{r}\frac{\partial \theta _{2}}{\partial r}+\frac{\partial ^{2}\theta _{2}}{\partial z^{2}}= \text {Re}\text {Pr}\bigg [-\frac{1}{r}\frac{\partial \psi _{0}}{\partial z} \frac{\partial \theta _{1}}{\partial r}+(\frac{1}{r}\frac{\partial \psi _{0} }{\partial r}+1)\frac{\partial \theta _{1}}{\partial z}\nonumber \\&\qquad -\frac{1}{r}\frac{\partial \psi _{1}}{\partial z}\frac{\partial \theta _{0} }{\partial r}+\frac{1}{r}\frac{\partial \psi _{1}}{\partial r}\frac{\partial \theta _{0}}{\partial z}\bigg ] \end{aligned}$$

77 $$\begin{aligned}&\frac{d{{p_{2}}}}{dz} =\frac{1}{r}\frac{\partial }{\partial r}(r{\tau _{2rz} })+\frac{\partial }{\partial z}({\tau _{1zz}})+\frac{A}{r}(\frac{\partial \phi _{2}}{\partial r}-\frac{\partial \psi _{2}}{\partial r})+\text {Gr}\theta _{2}\nonumber \\&\quad -\text {Re}\bigg [-\frac{1}{r}\frac{\partial \psi _{0}}{\partial z} \frac{\partial }{\partial r}(\frac{1}{r}\frac{\partial \psi _{1}}{\partial r})-\frac{1}{r}\frac{\partial \psi _{1}}{\partial z}\frac{\partial }{\partial r}(\frac{1}{r}\frac{\partial \psi _{0}}{\partial r})\nonumber \\&\quad +(\frac{1}{r}\frac{\partial \psi _{0}}{\partial r}+1)\frac{\partial }{\partial z}(\frac{1}{r}\frac{\partial \psi _{1}}{\partial r})+\frac{1}{r}\frac{\partial \psi _{1}}{\partial r}\frac{\partial }{\partial z}(\frac{1}{r} \frac{\partial \psi _{0}}{\partial r})\bigg ] \end{aligned}$$

78 $$\begin{aligned}&\tau _{1rr} =-2\frac{\partial }{\partial r}(\frac{1}{r}\frac{\partial \psi _{0}}{\partial z})+2\alpha _{1}\frac{\partial }{\partial r}(\frac{1}{r} \frac{\partial \psi _{0}}{\partial r})\frac{\partial }{\partial r}(\frac{1}{r}\frac{\partial \psi _{1}}{\partial r}) \end{aligned}$$

79 $$\begin{aligned}&\tau _{1\theta \theta } =\frac{-2}{r^{2}}\frac{\partial \psi _{0}}{\partial z} \end{aligned}$$

80 $$\begin{aligned}&\tau _{1zz} =\frac{2}{r}\frac{\partial ^{2}\psi _{0}}{\partial z\partial r}-2\alpha _{1}\frac{\partial }{\partial r}(\frac{1}{r}\frac{\partial \psi _{1} }{\partial r})\frac{\partial }{\partial r}(\frac{1}{r}\frac{\partial \psi _{0} }{\partial r}) \end{aligned}$$

81 $$\begin{aligned}&\tau _{2rz} =\frac{\partial }{\partial r}(\frac{1}{r}\frac{\partial \psi _{2} }{\partial r})-\frac{1}{r}\frac{\partial ^{2}\psi _{0}}{\partial z^{2}} +\alpha _{1}\Bigg [\frac{\partial }{\partial r}(\frac{1}{r}\frac{\partial \psi _{0}}{\partial r})\frac{1}{r}\frac{\partial ^{2}\psi _{1}}{\partial r\partial z} \end{aligned}$$

82 $$\begin{aligned}&\qquad +\frac{\partial }{\partial r}(\frac{1}{r}\frac{\partial \psi _{1}}{\partial r})\frac{1}{r}\frac{\partial ^{2}\psi _{0}}{\partial r\partial z}+\frac{\partial }{\partial r}(\frac{1}{r}\frac{\partial \psi _{0}}{\partial z} )\frac{\partial }{\partial r}(\frac{1}{r}\frac{\partial \psi _{1}}{\partial r})\nonumber \\&\qquad +\frac{\partial }{\partial r}(\frac{1}{r}\frac{\partial \psi _{1}}{\partial z})\frac{\partial }{\partial r}(\frac{1}{r}\frac{\partial \psi _{0}}{\partial r})-\frac{1}{r}\frac{\partial \psi _{0}}{\partial z}\frac{\partial ^{2}}{\partial r^{2}}(\frac{1}{r}\frac{\partial \psi _{1}}{\partial r})\nonumber \\&\qquad -\frac{1}{r}\frac{\partial \psi _{1}}{\partial z}\frac{\partial ^{2}}{\partial r^{2}}(\frac{1}{r}\frac{\partial \psi _{0}}{\partial r})+\frac{1}{r} \frac{\partial \psi _{0}}{\partial r}\frac{\partial ^{2}}{\partial r\partial z}(\frac{1}{r}\frac{\partial \psi _{1}}{\partial r})\nonumber \\&\qquad +\frac{1}{r}\frac{\partial \psi _{1}}{\partial r}\frac{\partial ^{2}}{\partial r\partial z}(\frac{1}{r}\frac{\partial \psi _{0}}{\partial r})\Bigg ] \end{aligned}$$

The boundary conditions are given be:

83 $$\begin{aligned}&\psi _{2}=0,\;\;\frac{\partial }{\partial r}(\frac{1}{r}\frac{\partial \psi _{2}}{\partial r})=0,\;\;\frac{\partial \theta _{2}}{\partial r}=0,\;\;\varphi _{2}=0\;\;\text {on}\;r=0 \end{aligned}$$

84 $$\begin{aligned}&\psi _{2}=F_{2},\;\;\frac{1}{r}\frac{\partial \psi _{2}}{\partial r}=0,\;\;\theta _{1}=0,\;\;\varphi _{2}=F_{s_{2}}\;\text {on}\;r=h \end{aligned}$$

These equations were solved numerically using DSolve of Mathematica software (https://www.wolfram.com/mathematica/new-in-11/). In the absence of heat transfer, we get the same solutions of stream functions for a second-grade fluid and dust particles as obtained by^28^. Also, if there is no solid particles and heat transfer, the solution is same in the paper^12^.

## Results and discussion

**Figure 2:**
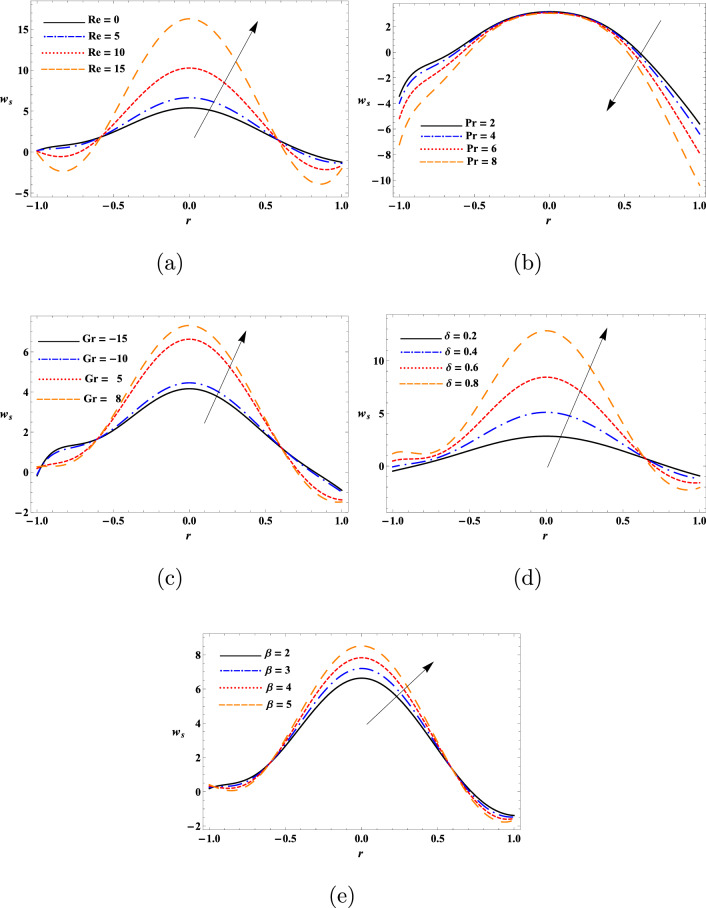
Variation of the dust particles velocity as a function of (**a**) $\text {Re}$, (**b**) $\text {Pr}$, (**c**) $\text {Gr}$, (**d**) $\delta$, and (**e**) $\beta$.

**Figure 3:**
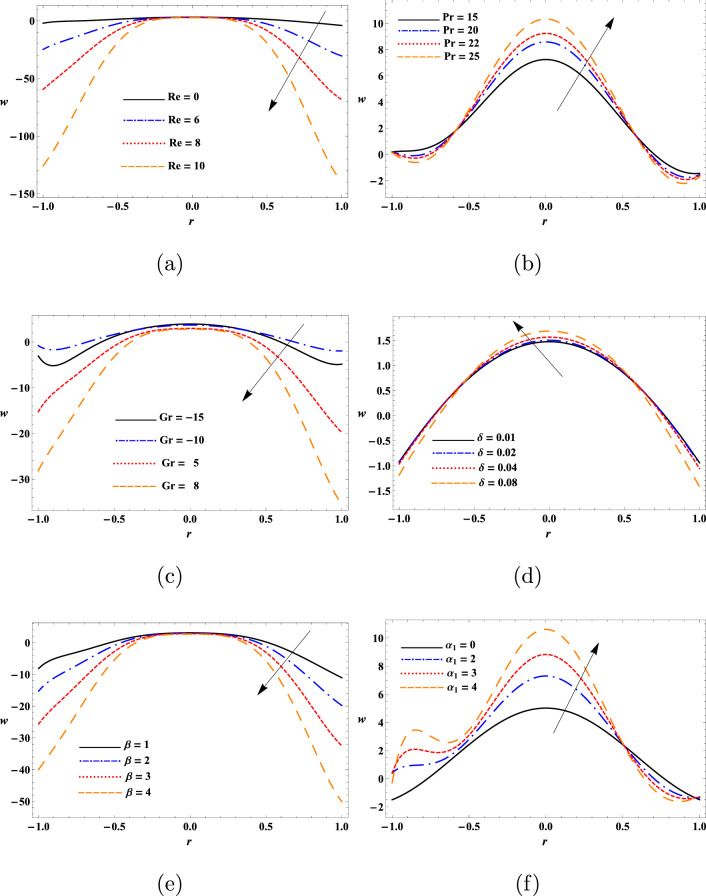
Variation of the fluid velocity as a function of (**a**) $\text {Re}$, (**b**) $\text {Pr}$, (**c**) $\text {Gr}$, (**d**) $\delta$, (**e**) $\beta$, and (**f**) $\alpha _{1}$.

**Figure 4:**
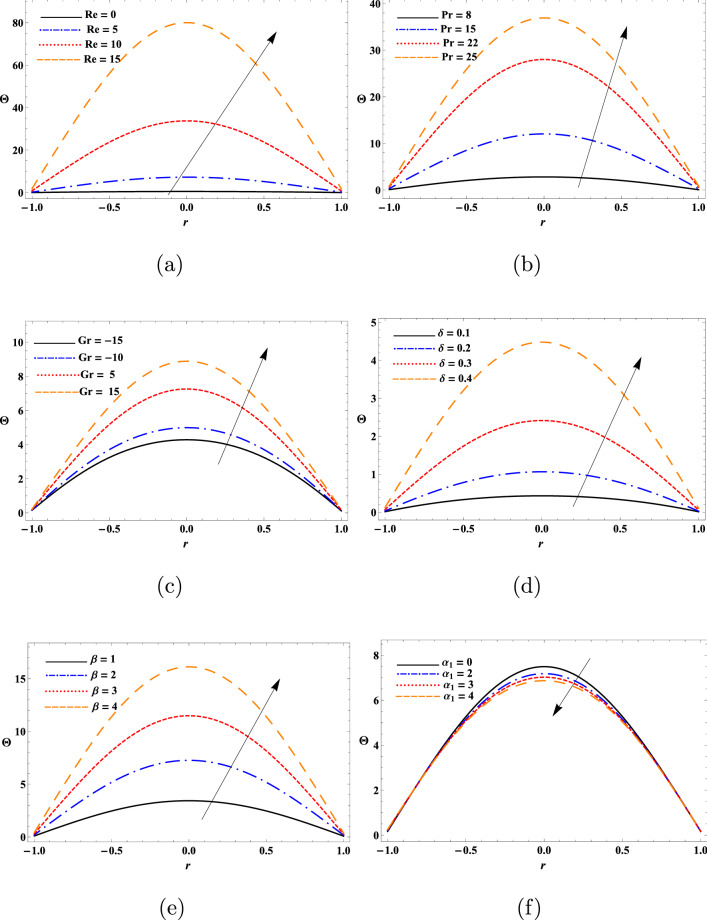
Variation of fluid temperature as a function of (**a**) $\text {Re}$, (**b**) $\text {Pr}$, (**c**) $\text {Gr}$, (**d**) $\delta$, (**e**) $\beta$, and (**f**) $\alpha _{1}$.

**Figure 5:**
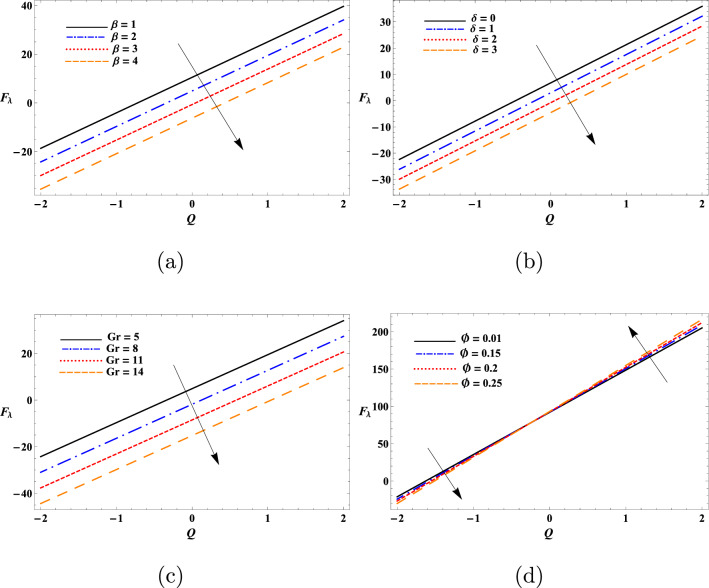
Variation of frictional force as a function of (**a**) $\beta$, (**b**) $\delta$, (**c**) $\text {Gr}$, and (**d**) $\phi$.

**Figure 6:**
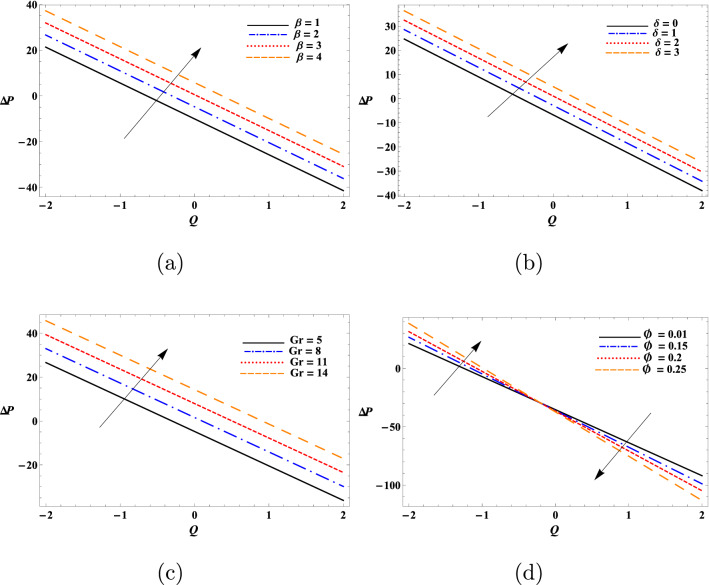
Pressure profiles of the fluid against (**a**) $\beta$, (**b**) $\delta$, (**c**) $\text {Gr}$, and (**d**) $\phi$.

**Figure 7:**
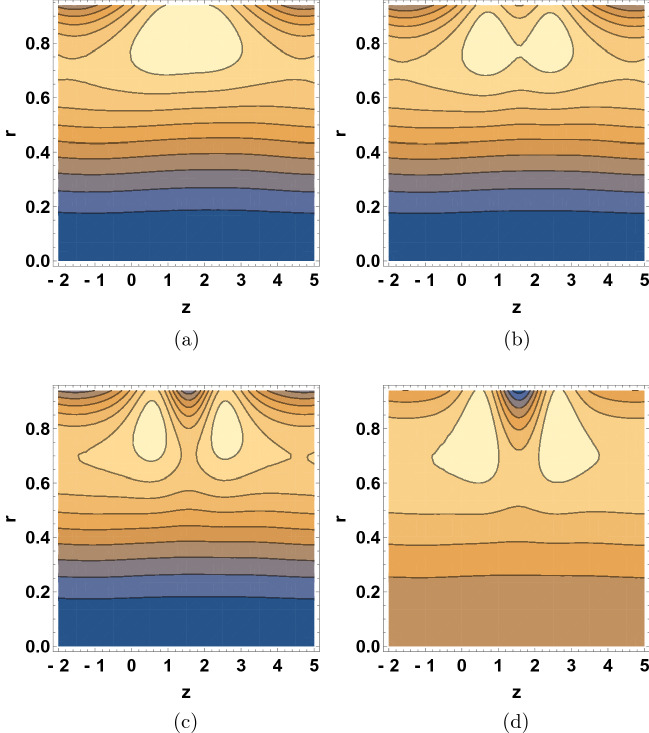
Streamline patterns of the fluid for (**a**) $\beta =1$, (**b**) $\beta =2$, (**c**) $\beta =3$ and (**d**) $\beta =4$.

**Figure 8:**
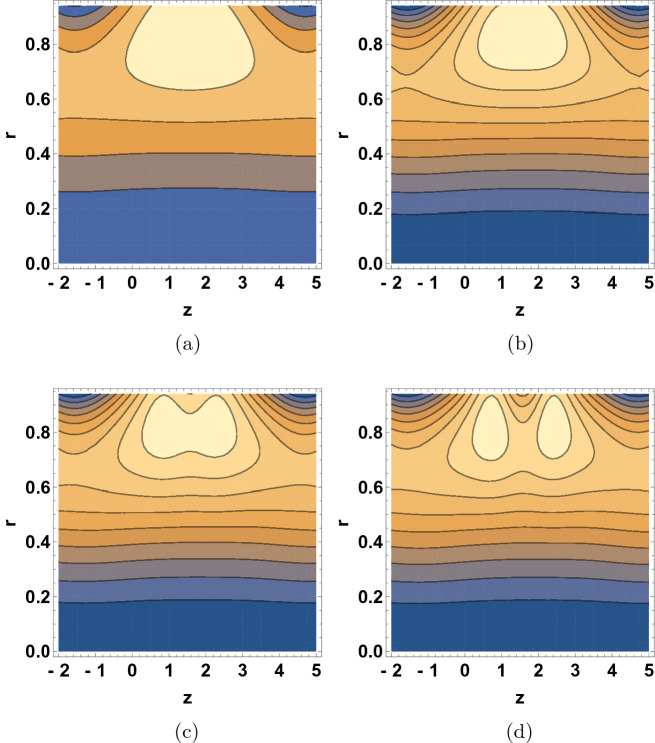
Streamline patterns of the fluid for (**a**) $\delta =0$, (**b**) $\delta =0.01$, (**c**) $\delta =0.02$, and (**d**) $\delta =0.03$.

**Figure 9:**
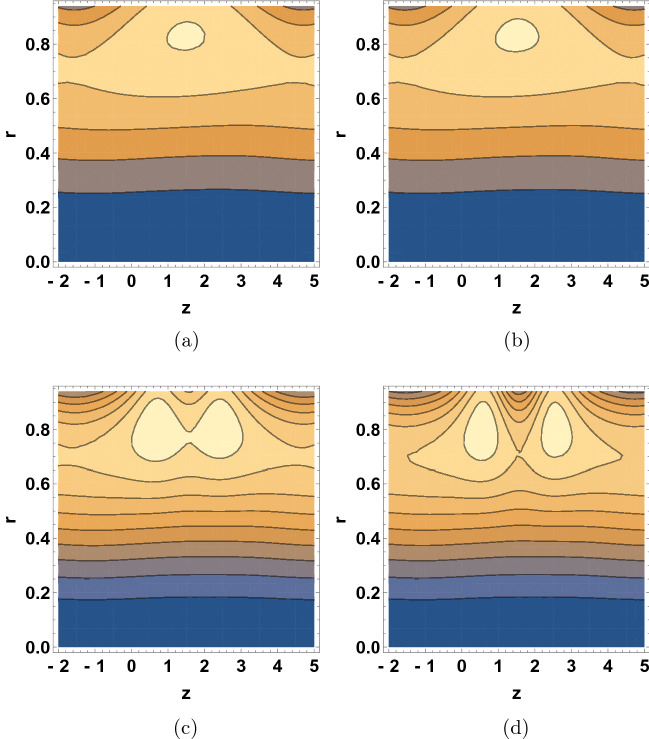
Streamline patterns of the fluid for (**a**) $\text {Re}=0$, (**b**) $\text {Re}=2$, (**c**) $\text {Re}=5$, and (**d**) $\text {Re}=6$.

**Figure 10:**
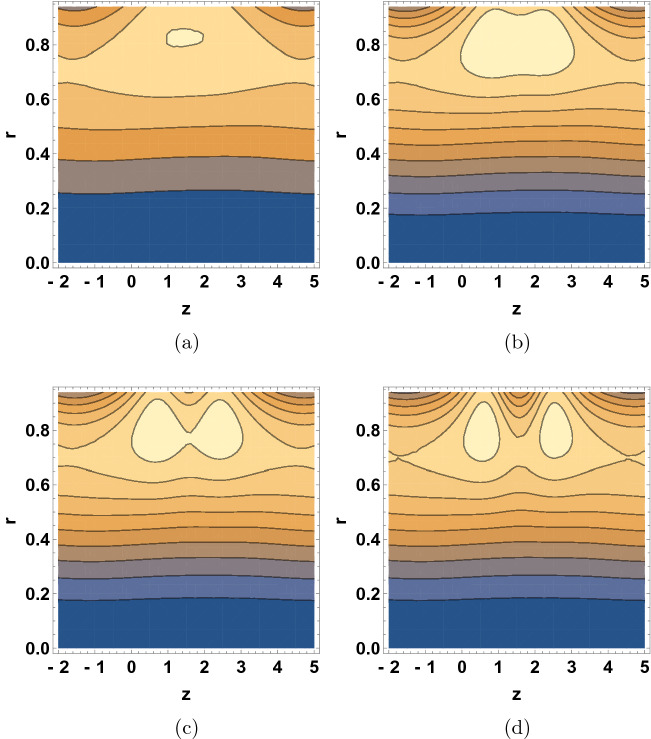
Streamline patterns of the fluid for (**a**) $\text {Pr}=8$, (**b**) $\text {Pr}=10$, (**c**) $\text {Pr}=12$, and (**d**) $\text {Pr}=14$.

**Figure 11:**
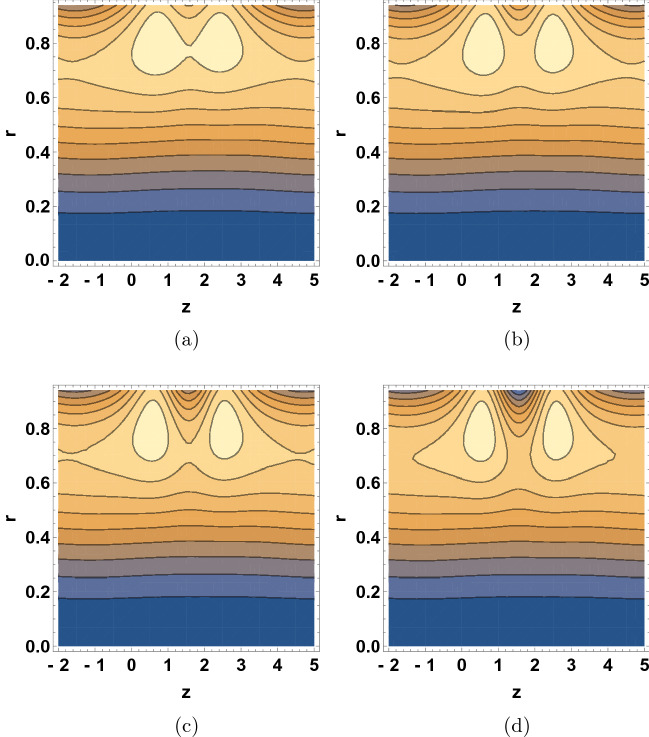
Streamline patterns of the fluid for (**a**) $\text {Gr}=5$, (**b**) $\text {Gr}=6$, (**c**) $\text {Gr}=7$, and (**d**) $\text {Gr}=8$.

This study aimed to determine the influence of heat transfer on the transport behaviours of a second-grade dusty fluid flown in a flexible tube with peristaltic movement of the wall. The motion of dust particles and fluid were analytically modeled diverse nonlinear equations and solved analytically using perturbation approach. Numerical solutions of these modeled equations were obtained by DSolve of the Mathematica 11 software (https://www.wolfram.com/mathematica/new-in-11/). Relevant parameters associated to the impact of heat transfer on the peristaltic transport of the fluid like $\text {Re}$, $\text {Pr}$, $\text {Gr}$, $\delta$, $\beta$ and $\alpha _{1}$ were evaluated to determine the mechanism of such transport. In addition, diverse physical quantities such as $w_{s}$, *w*, $\theta$, $F_{\lambda }$, $\Delta {p}$ and streamline pattern were determined to describe the transport process. Figures 2, 3, 4, 5, 6, 7, 8, 9, 10 and 11 illustrates the variation of the dust particles velocity, fluid velocity, temperature, frictional force, pressure rise, and trapping.

The values of $\text {Re}$, $\text {Pr}$, $\text {Gr}$, $\delta$, and $\beta$ were shown to significantly influence dust particles velocity during the fluid transport (Fig. 2). With the increase of $\text {Re}$, $\text {Gr}$ and $\beta$ values, the velocity of the parcicles was first reduced in the wave number range of $-1.0\le r\le -0.6$ and $0.6\le r\le 1.0$ then increased in the range of $-0.6\le r\le 0.6$, This increase refers that viscous forces are weakening thus particles may flow more smoothly. Furthermore, the velocity of the particles was decreased with the increase of Prandtl number in the range of $-1.0\le r\le 1.0$ and the particles velocity was first increased with the increase of wave number $\delta$ in the range of $-1.0\le r\le 0.6$ and then dropped in the range of $0.6\le r\le 1.0$. It is observed that the dust particles velocity is maximum in the central line of the artery for all the four values of $\text {Re}$, $\text {Pr}$, $\text {Gr}$, $\delta$, and $\beta$ and therefore, maximum decrease in the central axis of the tube. The present observation are with the findings of^28^.

The results in Fig. 3 show the parabolic profile of the fluid velocity at the inlet $r=0$ of the tube for constant parameter’s values. The fluid velocity was found to decrease with the increase of $\text {Re}$ and $\beta$ in the range of $-1.0\le r\le -0.3$ and $0.3\le r\le 1.0$. However, the fluid velocity remained insensitive to $\text {Re}$ and $\beta$ in the range of $-0.3\le r\le 0.3$. As decrease in Reynolds number enhances the friction force thus causing reducing in the fluid velocity. The fluid velocity was increased with the increase of $\text {Pr}$ and wave number in the range of $-0.6\le r\le 0.6$, so the fluid flows more smoothly and efficiently in the desired direction. The fluid velocity was dropped with the increase of $\text {Pr}$ and wave number ($\delta$) in the tube walls viscinity. With the rise in the Prandtl number (Pr), we see an increasing manner in the velocity in the center of the tube. Furthermore, The fluid velocity was reduced with the increase of $\text {Gr}$ and increased with the increase of $\alpha _{1}$. One can observe that fluid velocity is in oscillatory behavior, which may be due to peristalsis. Various physical parameters were shown appreciably affect the temperature profile of the fluid (Fig. 4). The temperature being the mean molecular kinetic energy of the fluid depends on the particles velocity. In this study, the values of $\theta$ was increased with the increase of $\text {Re}$, $\text {Pr}$, Gr, $\delta$ and $\beta$, whereas it was dropped with the increase of $\alpha _{1}$ in the proximity of the tube surface *r*. In short, the temperature variation of the fluid at the inlet $r=0$ was discerned to be parabolic for constant values of the physical parameters. This result is in good agreement with the results obtained by^15^. From the observations of the results, it has been noted that parameters involved have a similar role in the temperature, since the temperature determines the average kinetic energy which is related to the motion of fluid particles.

Figure 5 displays the frictional force profiles of the fluid for various values of $\beta$, $\delta$, $\text {Gr}$, and $\phi$. The values of $F_{\lambda }$ were decreased rapidly with increasing values $\beta$, $\delta$ and $\text {Gr}$, while it was increased with the increase of volume flow rate ($\nonumber Q$). Furthermore, the values of $F_{\lambda }$ were decreased for $\nonumber Q\in (-2,0)$ and increased for $\nonumber Q\in (0,2)$ with the increase of $\phi$.

Figure 6 depicts variation of the dusty fluid pressure for various values of $\beta$, $\delta$, $\text {Gr}$, and $\phi$. The values of $\Delta {p}$ of the fluid were increased rapidly with the increase of $\beta$, $\delta$ and $\text {Gr}$. In addition, $\Delta {p}$ values of the fluid were increase with the increase of $\phi$ for $\nonumber Q\in [-2,0]$ and it decreases for $\nonumber Q\in [0,2]$. The variation of $\Delta {p}$ with respect to the studied parameters of the dusty fluid followed a reverse trend compared to $F_{\lambda }$. As expected, the pressure rise results in higher values for small mean volume flow rates and lower values for large $\nonumber Q$. Furthermore, peristaltic pumping takes place in this area $-2\le \nonumber Q\le 2$, otherwise augmented pumping occurs.

Figure 7 illustrates the effect various $\beta$ values on the streamline patterns of the dusty-fluid. It is important to mention that the study of trapping phenomena in the field of peristaltic transport of fluid remains challenging wherein streamline patterns of the dusty-fluid can provide valuable insight. In the wave frame, the streamline patterns are usually comparable to the boundary wall. However, under certain situations the streamlines of the fluid split to entrap a bolus and shoved together with the peristaltic wave at the wave speed. The sizes of the trapped bolus were reduced with the increase of $\beta$ values and then gradually disappeared at large values of $\beta$, the movement of bolus can be seen stretching towards the upward direction.

Figure 8 shows the $\delta$ dependence of the streamline patterns of the fluid motion wherein the trapping occurred close to the tube boundary. The sizes of the bolus were decreased with the increase of $\delta$ values. As wave number is raised, the movement of bolus divided into two small bolus and the streamlines increase. Figure 9 depicts the $\text {Re}$ dependent variation in the streamline patterns of the fluid. Yet again, the sizes of the trapped bolus were enlarged with the increase of Reynolds number, viscous force weakens thus it was observed that the motion of the fluid gets smoother and bolus expands and move towards upward direction. Figure 10 demonstrates the alteration in the streamline hallucinations for different values of $\text {Pr}$. The streamlines of the fluid were significantly influenced by the variation of $\text {Pr}$ where in the volume of bolus was remarkably enhanced. Figure 11 displays the dependence of the fluid’s streamline patterns on the variation of $\text {Gr}$. The number of bolus was dropped and the size was enlarged with the increase of $\text {Gr}$, indicating the trapping of bolus bounded by invariant closed streamlines of the dusty fluid. As the values of Pr and Gr are increased, a clear change in the formation and volume of the bolus was observed. The motion of the fluid particles is towards the direction of the Prandtl and Grashof numbers.

## Conclusion

This paper comprehensively determined the effects of heat transfer on the transport features of a second-grade dusty fluid flown in a flexible tube under peristaltic motion of the wall for the first time. Both fine dust particles and fluid motion through the cylindrical tube were modeled using the nonlinear coupled differential equations. Standard perturbation method was used to get the analytical solutions of the model equations. Based on the numerical results obtained using DSolver of the Mathematica software the following conclusions may be drawn:

1. With the increase of $\text {Re}$ and $\text {Gr}$ values, the trapped bolus of dust particles and fluid were enlarged due to viscous force weakens thus causing the motion of the fluid gets smoother and bolus get enhanced.
2. The temperature of the dusty fluid was increased with the increase of $\text {Re}$, $\text {Pr}$, Gr, $\delta$ and $\beta$ values, the fluid flows more smoothly and efficiently in the desired direction.
3. The velocity of the dust particles and fluid was increased with the increase of $\text {Pr}$ and wave number in the range of $-0.6\le r\le 0.6$. One can observe that the velocity is in oscillatory behavior, which may be due to peristalsis.
4. The parameters $\text {Re}$, $\text {Pr}$, Gr, $\delta$ and $\beta$ have a tendency to speed up the motion of fluid. It is revealed that up to $10\%$ fluid velocity increasing occurs in the presence of these parameters.
5. The frictional forces in the fluid showed reverse trend than the pressure rise.
6. Generally, the heat transfer-mediated peristaltic transport properties of the proposed dusty fluid were affirmed to depend considerably on various physical parameters.
7. The achieved results were in good agreement with the recent state-of-the-art works reported in the literatures.
8. It was asserted that the present findings may be beneficial for the advancement of fluid mechanics, biomendical sciences, and engineering.
9. The generated new knowledge can certainly help the surgeon to control the gastric fluid flow in small intestine during endoscopy.

### Future directions

In future one should exploit both deterministic and stochastic numerical solver for both fluid mechanics problems of paramount significance. In future, one may implement the Lobatto IIIA scheme for the numerical treatment of many potential applications arising in the fields of Bioinformatics, astro/plasma/atomic physics, nonlinear circuit models, fluid mechanics, financial mathematics and COVID-19 virus models.

## Data Availability

The datasets generated and/or analyzed during the current study are not publicly available due [All the required data are only with the corresponding author] but are available from the corresponding author on reasonable request.

## List of symbols

*a*: Tube radius
*b*: Wave amplitude
$$\lambda$$: Wave length
*c*: Wave speed
$$\mu$$: Viscosity coefficient
*N*: Number density of solid particles
*K*: Stokes resistance coefficient
$\alpha$: Thermal expansion coefficient
$$c_{p}$$: Specific heat
$$\kappa$$: Thermal conductivity
$Q_{0}$: Constant heat addition/absorption
$\alpha _{1}$: Second grade parameter
*Re*: Reynolds number
*Pr*: Prandtl number
*Gr*: Grashof number
$\beta$: Heat source/ sink parameter
$\delta$: The wave number
$\phi$: Wave amplitude ratio
$\psi$: Stream function for dust fluid
$\varphi$: Stream function for solid particles
$$\theta$$: Dimensionless temperature
$\Delta {p}$: The pressure rise
$F_{\lambda }$: The friction force
